# A Rapid Localization Method Based on Super Resolution Magnetic Array Information for Unknown Number Magnetic Sources

**DOI:** 10.3390/s24103226

**Published:** 2024-05-19

**Authors:** Linliang Miao, Tianyi Zhang, Chao Zuo, Zijie Chen, Xiaofei Yang, Jun Ouyang

**Affiliations:** 1 School of Integrated Circuits, Huazhong University of Science and Technology, Wuhan 430074, China; mll@hust.edu.cn (L.M.); m202272787@hust.edu.cn (T.Z.); m202272796@hust.edu.cn (Z.C.); yangxiaofei@hust.edu.cn (X.Y.); 2 Hubei Key Laboratory of Marine Electromagnetic Detection and Control, Wuhan 430064, China; 3 Wuhan Second Ship Design and Research Institute, Wuhan 430064, China

**Keywords:** magnetic localization, multi-target localization, super resolution, trust region reflective

## Abstract

A rapid method that uses super-resolution magnetic array data is proposed to localize an unknown number of magnets in a magnetic array. A magnetic data super-resolution (SR) neural network was developed to improve the resolution of a magnetic sensor array. The approximate 3D positions of multiple targets were then obtained based on the normalized source strength (NSS) and magnetic gradient tensor (MGT) inversion. Finally, refined inversion of the position and magnetic moment was performed using a trust region reflective algorithm (TRR). The effectiveness of the proposed method was examined using experimental field data collected from a magnetic sensor array. The experimental results showed that all the targets were successfully captured in multiple trials with three to five targets with an average positioning error of less than 3 mm and an average time of less than 300 ms.

## 1. Introduction

Magnetic positioning techniques play a vital role in applications such as precision industrial control, intrusion target detection, and medical intervention [1,2,3,4,5,6,7,8]. In industry, magnetic positioning can improve the positioning accuracy of equipment such as automated guided vehicles [1]. In the medical field, magnetic positioning techniques have been used to track the motion of human organs such as fingers [3], tongues [4], and heart valves [5], as well as medical devices such as wireless capsules [6,7] and interventional catheters [8]. The two main types of magnetic sources generating magnetic fields are permanent magnets and electromagnetic coils. Compared with electromagnetic localization methods [9], permanent magnet-based methods do not require an external power supply, which makes it easier to achieve passive, wireless, and less invasive localization schemes.

In the application of permanent-magnet localization, the technique of localizing a single magnet using a stationary magnetic array is well established. The position and orientation of the permanent magnets are generally estimated by building an optimization model of the target parameters based on a dipole model, measuring the magnetic field using an array of magnetic sensors, and using an optimization algorithm to minimize the cost function [10]. In addition, studies have been conducted on target position inversion tracking using recursive Bayesian estimation [11] or artificial neural networks [12,13].

Currently, researchers are focusing on the inverse problem of multiple magnets in a magnetic array. The primary difficulty in multi-magnet localization is that the increased number of magnets introduces more parameters to be solved. Clearly, a dimensional explosion of the solution space leads to a long or even unsolvable solution time for this problem. Researchers have conducted numerous optimization studies on parameter-solving methods. Song et al. [14] encapsulated two orthogonal permanent magnets in a wireless capsule, applied particle swarm optimization and the Levenberg-Marquardt algorithm (PSO-LM) to compute the 5D position of each magnet, and implemented a three-magnet localization system. Yang et al. [15] tracked three cylindrical permanent magnets using a hybrid algorithm consisting of the PSO, cloning, and LM algorithms. The average positional accuracy of tracking the three magnets in real-time was 3.7 mm over the delimited area. Cameron et al. [16] derived a gradient analytical formulation to track up to four magnets simultaneously with high accuracy and low computational latency. Lv et al. [17] implemented three-magnet localization based on individual memory whale optimization and the Levenberg-Marquardt algorithms (IMWO-LM) to achieve a positional accuracy of 3.46 mm. In addition, some applications, such as intrusion target detection, lack important information regarding the number of targets. Multi-magnet inversion in this application is more difficult; however, there are few studies on the inversion of an unknown number of multi-magnets. Chang et al. [18] located the position of an unknown number of multiple magnetic dipoles by including the number of targets as part of the parameter optimization. However, this method requires appropriate initialization conditions, and there is still room for improvement in the accuracy of target number inversion. Overall, there are still three problems with multi-magnet inversion in arrays: (1) conventional model inversion is difficult when the number of targets is unknown; (2) a larger number of targets leads to a higher dimension of the solution space, the conventional global search algorithms are prone to falling into the local optimum, and the accuracy of the inversion decreases or even fails to be inverted; and (3) the computation time of the iterative optimization inversion method is positively correlated with the number of targets, which affects the real-time performance of the array system.

A common way to address this problem is to fully exploit prior knowledge to reduce the solution space range or compress the solution space dimension. Similar approaches have emerged for some nonarray magnet localization applications, such as unexploded ordnance (UXO) detection and underground deposit detection. In these applications, magnetic sensors perform mobile measurements to acquire denser grid data than array measurements. By analyzing the dense magnetic vector or tensor grid data in combination with certain magnetic field eigenvalues, it is possible to quickly determine the position of the target or directly determine all the magnet parameters. Li et al. [19] used the magnetic gradient tensor Helbig integral to estimate the horizontal position of the target and used the magnetic vector and tensor field data directly above the source to calculate the depth of the source and magnitude of the dipole moment. This method improves the localization accuracy by more than a factor of 5 over the classical method. Ding et al. [20] used the tilt angle to determine the number of sources when processing magnetic gradient tensor data, used the rotationally invariant normalized source strength (NSS) to estimate the approximate horizontal coordinates of the sources, and ultimately used the differential evolution (DE) algorithm to estimate the position and magnetic moment of the sources. Li et al. [21] used an improved target area identification tilt angle and self-adaptive fuzzy c-means (SAFCM) clustering algorithms to process grid measurement data of magnetic gradient tensors, which can accurately identify the two-dimensional (2D) region boundaries of objects with different burial depths. Zheng et al. [22] fused the Eulerian method and DBSCAN algorithms to invert the UXO position for UXO detection, which could accurately locate multiple targets on the subsurface. Because of the high-density magnetic data obtained from mobile measurements, the above methods can quickly obtain the parameter information of multiple targets, and the performance of some of the methods is not affected by an increase in the number of targets. This makes it more advantageous for multitarget localization, and the number of magnets that can be located simultaneously far exceeds that of the array measurement methods. However, scanning grid measurements exhibit poor real-time performance and are unsuitable for applications, such as medical device positioning.

To integrate the advantages of the high information density of mobile measurements and real-time performance of array measurements, the spatial resolution of the array measurement system needs to be improved. Improving the resolution by increasing the sensor density increases the difficulty and cost of hardware implementation. Therefore, the application of super-resolution algorithms to improve the resolution of magnetic sensor arrays was considered. Super-resolution techniques have a wide range of applications in the imaging field and can reconstruct low-resolution (LR) images into clear, high-resolution (HR) images. Super-resolution methods can be divided into interpolation-, reconstruction-, and learning-based methods. In recent years, deep learning has become the primary method for image super-resolution. Networks such as SRCNN [23], which use three convolutional layers, and networks such as EDSR [24], which include residual structures, have significantly improved the quality of reconstructed images. Networks based on attention mechanisms, such as SAN [25] and RCAN [26], have achieved better results. Currently, NAFSR [27] and SwinIR [28] are the main state-of-the-art networks in the field of image super-resolution. The former constructed a low-complexity convolutional neural network without an activation function, whereas the latter constructed a neural network with a transformer structure. Both achieved remarkable results in super-resolution tasks. These neural-network-based super-resolution methods have also been used in a wide range of applications ranging from astrophysics to seismic data analysis. However, their application in the enhancement of magnetic array data has rarely been explored. In this study, we apply these methods to magnetic array data enhancement.

In this paper, a multi-magnet localization method using super-resolution magnetic array information is proposed. To address the problem of an unknown number of targets, a deep super-resolution neural network without an activation function was used to enhance the 8 × 8 sparse array data into 64 × 64 high-resolution data. The number of targets was determined by calculating the high-resolution NSS. Normalized source intensities were used to estimate rough horizontal positions. The target heights were then calculated based on magnetic tensor inversion near the horizontal position. Obtaining the approximate 3D position of the target can significantly reduce the difficulty of solving the subsequent optimization algorithm and prevent falling into a local optimum. To address the problem of long computation time, a trust region reflective optimization algorithm with a rough initial positioning value was used to achieve fast computation of the target parameters. This part abandons the conventional heuristic optimization method, which improves computational speed while guaranteeing inversion accuracy.

## 2. Methodology

**Figure 1:**
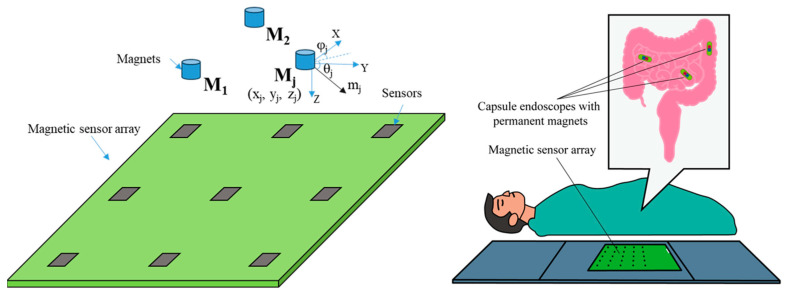
Magnetic sensor array, magnetic dipole model and medical applications such as capsule endoscopic positioning in digestive tract.

In general, magnetic target inversion is used to estimate the location and magnetic moments of magnetic anomalies in a region consisting of magnetic fields. When the magnetisation intensity is fairly uniform and the distance between the source centre and the observation point is more than three times the geometrical length of the target, the magnetic source can be considered a magnetic dipole [10]. As shown in Figure 1, the magnetic dipole source properties can be described in terms of six degrees of freedom, divided into two categories, (a) three of which describe the position, usually expressed in terms of horizontal position (*x* and *y*) and height (*z*), and (b) the other three describe the magnetic moments, usually expressed in terms of the magnitude of the moments (*M*), the inclination of the magnetisation (*θ*) and the magnetic declination of the magnetisation (*φ*). Thus, the inversion of multiple magnets is to estimate the values of the six elements of the multiple magnetic dipoles.

The $k_{th}$ magnetic sensor measurement in the array is the superposition of the vector magnetic field generated by *N* magnets at the sensor position. The triaxial magnetic field of the sensor is given by Equation (1).

(1) $$\begin{array}{l} B_{x,k}=\sum_{j=1}^{N}\frac{\mu_{0}}{{4\pi r_{j,k}}^{5}}\left[3\left(\vec{r_{j,k}}\cdot \vec{m_{j}}\right)\left(x_{k}-x_{j}\right)-{r_{j,k}}^{2}m_{j}\cos\theta_{j}\cos\varphi_{j}\right]\\ B_{y,k}=\sum_{j=1}^{N}\frac{\mu_{0}}{{4\pi r_{j,k}}^{5}}\left[3\left(\vec{r_{j,k}}\cdot \vec{m_{j}}\right)\left(y_{k}-y_{j}\right)-{r_{j,k}}^{2}m_{j}\cos\theta_{j}\sin\varphi_{j}\right]\\ B_{z,k}=\sum_{i=1}^{N}\frac{\mu_{0}}{{4\pi r_{j,k}}^{5}}\left[3\left(\vec{r_{j,k}}\cdot \vec{m_{j}}\right)\left(z_{k}-z_{j}\right)-{r_{j,k}}^{2}m_{j}\sin\theta_{j}\right] \end{array}$$

where $\left(m_{j},\theta_{j},\varphi_{j}\right)$ are the magnetic moment parameters of the j target, $r_{j,k}$ is the position vector from the j target to the k magnetic sensor [13], and $\left(x_{j},\;y_{j},z_{j}\right)$ and $\left(x_{k},\;y_{k},z_{k}\right)$ are the 3D coordinates of the jth target and the k sensor, respectively. The parameters to be inverted are $\left(x_{j},\;y_{j},z_{j},m_{j},\;\theta_{j},\varphi_{j}\right),\;j=1,2,\ldots ,N$. Obviously, the complexity of parameter inversion rises as the number of magnets *N* increases.

### 2.1. Proposed Method

**Figure 2:**
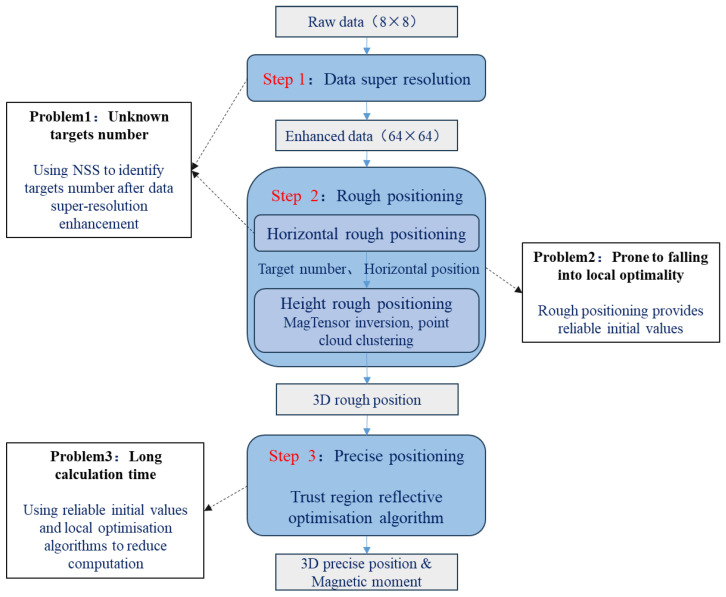
Method overview.

The method proposed in this study is applicable to the inversion of multi-magnet parameters for array magnetic detection. The method consists of three steps, and its overall concept is illustrated in Figure 2. First, the resolution of the magnetic array data is enhanced. We developed a super-resolution method for magnetic field data based on an activation-free function deep neural network that can obtain high-resolution magnetic vector data and further compute magnetic tensor data. In the second step, super-resolution magnetic field data are used to invert and obtain the 3D rough localization results. In this step, the number and horizontal position of the targets are obtained using NSSs. The target point cloud is inverted based on the magnetic tensor, and the target height is roughly determined after clustering. In the third step, based on the approximate data obtained in the previous step, a local optimization algorithm is used to quickly search for the precise location of the target and the magnetic moment parameters. The algorithm using a super-resolution magnetic gradient tensor and TRR optimization algorithm is subsequently referred to as SRMGT-TRR.

### 2.2. Magnetic Field Super-Resolution Neural Network

High-resolution magnetic field data can help achieve a more accurate target number identification and rough target localization. The goal of super-resolution magnetic data is to develop an end-to-end mapping function *F* that reconstructs the high-resolution magnetic array data $B_{m}^{HR}$ from the input magnetic array data $B_{m}^{LR}$. If the training dataset is ${\left\{B_{m}^{LR},B_{m}^{HR}\right\}}_{m=1}^{U}$, then the mapping function must address the following issues:

(2) $$\hat{\delta }=\arg\underset{\delta }{\min}\frac{1}{U}\overset{U}{\underset{m=1}{\sum }}Loss^{SR}\left(F_{\delta }\left(B_{m}^{LR}\right),B_{m}^{HR}\right)$$

where *U* is the total number of data records, *δ* is the weight value of the mapping function, and Loss is the loss function, which measures the deviation between the reconstructed array data and the original high-resolution data.

**Figure 3:**
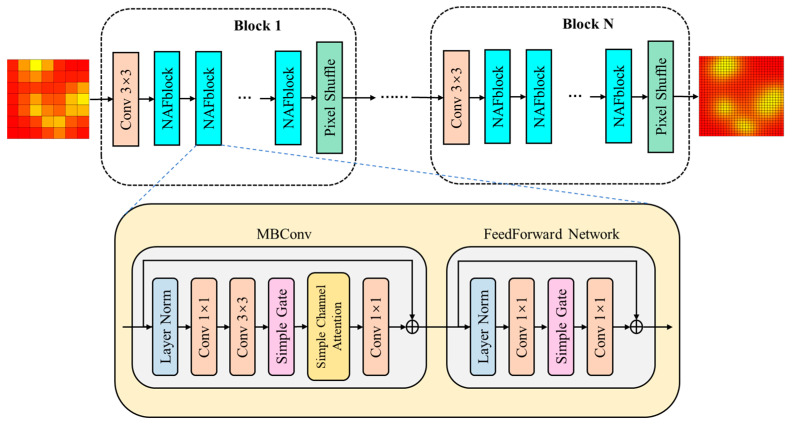
Overall structure of the proposed magnetic super-resolution network.

In this study, we built a deep magnetic data super-resolution network without any activation functions, as shown in Figure 3. This reduces the computational requirements while maintaining optimal performance. The backbone of the network consists of nonlinear activation-free (NAF) blocks and PixelShuffle upsampling modules [27], which enable a super-resolution performance that exceeds that of other methods while reducing computational effort. The network employs a progressive upsampling architecture, in which multiple modules are stacked to gradually increase the resolution of the magnetic array data.

**Figure 4:**
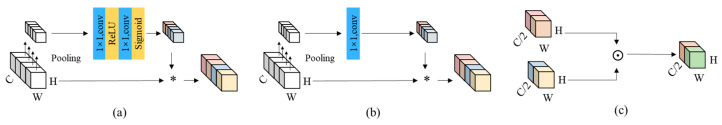
Illustration of (**a**) channel attention (CA), (**b**) simplified channel attention (SCA), and (**c**) simple gate (SG). ⊙/∗: element-wise/channel-wise multiplication.

The basic function of the NAF module is to extract input image features using a stacked layer normalization module, convolution module, simple gate (SG) module, and simple channel attention (CA) module. As shown in Figure 4, conventional CA is averaged over each channel of the input feature map (C × H × W) to obtain a set of vectors. The two input convolutions are then passed through an activation function to obtain a weight vector, and the weights are multiplied by the input features to obtain new features weighted by the channel dimensions. Owing to the correlation between the different measurement axes of the magnetic sensor, the use of CA helps to extract the interaction information between the data channels of the magnetic array. Simple CA (SCA) cancels the convolution and activation functions in the middle 2 layers of the original CA and replaces them with a 1 × 1 convolution operation. An SG divides the input of C × H × W into two equal parts C/2 × H × W, multiplies them to obtain a new feature, and uses this to replace the activation function. In [27], it was verified that this simplification has little or no effect on network performance but effectively reduces the number of operations.

The *SCA* and *SG* mechanisms are shown in Equation (3).

(3) $$\begin{array}{l} SCA\;\left(X\right)=X*\left(W_{2}^{\;}\max\left(0,W_{1}^{\;}pool\left(X\right)\right)\right)\\ SimpleGate(X,\;Y)=X\;⊙Y \end{array}$$

where *SCA* denotes the simplified channel attention, *X* denotes the feature map, and pool denotes the global maximum pooling operation. *W*_1_ and *W*_2_ are learnable matrices. Finally, * denotes per-channel product operation.

Upsampling was implemented using the PixelShuffle module. This upsamples a low-resolution image to a high-resolution image by learning the mapping relationship between low-resolution and high-resolution images. First, it learns a residual image by nonlinearly transforming an input low-resolution image. The residual image is then summed pixel-by-pixel with the original low-resolution image to obtain the final high-resolution image. Compared to conventional interpolation methods, PixelShuffle has better reconstruction results and higher computational efficiency.

The current mainstream framework for super-resolution is the post-upsampling framework, which is computationally small and can complete upsampling in one step. However, the magnification of magnetic array data enhancement is generally high, and the post-upsampling framework increases the learning difficulty of the upsampling factor. On the other hand, the progressive upsampling framework gradually increases the resolution of the array image, and the learning process is smoother. Therefore, the network designed in this study adopted a progressive upsampling architecture. Each module takes the low-resolution feature maps of the previous stage as input and outputs two high-resolution feature maps. Each module first connects multiple NAF blocks in series for feature extraction, then inputs the extracted features and low-resolution maps from the initial input of the module into the upsampling module, and outputs the high-resolution results.

### 2.3. 3D Rough Positioning

Conventional optimization algorithms yield satisfactory results when locating 1–3 targets based on magnetic arrays. However, as the number of targets continues to increase, the dimensions of the parameters to be solved expand, and a simple optimization algorithm is difficult to handle. To constrain the solution range of the optimization algorithm, it is necessary to obtain more a priori information based on enhanced magnetic field data.

2D magnetograms obtained from sensor arrays have made it possible to obtain the rough horizontal positions of targets. However, the use of the peak of the total magnetic field to identify the number and horizontal position of targets has two drawbacks: (a) the peak of the total magnetic field and horizontal coordinates of the target do not necessarily coincide, and (b) the three-fold decaying property of the magnetic field with distance causes the magnetic fields generated by dipoles in close proximity to superimpose on each other.

**Figure 5:**
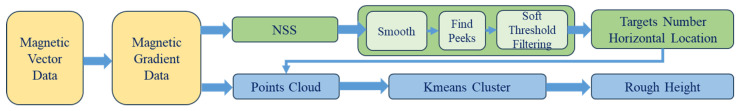
The process of 3D Rough Positioning.

As shown in Figure 5, after augmenting the array data into dense data, feature quantities, such as the magnetic gradient tensor, can be calculated relatively accurately from the 2D magnetic vector data. The magnetic gradient values are calculated using the four nearest data points in the *X* and *Y* directions of the position to be calculated. The NSS, which is unaffected by the direction of the dipole magnetic moment, can be calculated based on the magnetic gradient tensor and other characteristics. The magnetic gradient tensor [29] *G* is expressed as:

(4) $$G=\left[\begin{array}{ccc} g_{xx} & g_{xy} & g_{xz}\\ g_{yx} & g_{yy} & g_{yz}\\ g_{zx} & g_{zy} & g_{zz} \end{array}\right]=\left[\begin{array}{c} v_{1}\\ v_{3}\\ v_{2} \end{array}\right]\left[\begin{array}{ccc} \lambda_{1} & & \\ & \lambda_{3} & \\ & & \lambda_{2} \end{array}\right]{\left[\begin{array}{c} v_{1}\\ v_{3}\\ v_{2} \end{array}\right]}^{-1}$$

where $g_{ij},i=x,y,z,\;j=x,y,z$ are the elements of the magnetic gradient tensor and λ_1_, λ_2_, and λ_3_ represent the eigenvalues of *G*, and *v*_1_, *v*_2_,and *v*_3_ are the eigenvectors corresponding to the eigenvalues. The NSS can be obtained by solving the eigenvalue operation of the tensor matrix [21], as shown in Equation (5).

(5) $$\mathrm{NSS}=\sqrt{-\lambda_{3}^{2}-\lambda_{1}\lambda_{2}}\propto \frac{M}{r^{4}},\;\lambda_{1}\leq \lambda_{3}\leq \lambda_{2}$$

The magnitude of the NSS is proportional to the magnetic moment modulus and inversely proportional to the fourth power of distance. Therefore, the NSS is largest directly above the target and decays more rapidly away from the target. This reduces the effects arising from magnetic field overlap when the dipoles are closely spaced. The number of magnetic dipoles and the horizontal position can be identified by smoothing the NSS to find possible peaks and then using a soft threshold to filter reliable peaks. The threshold in this paper is set to the mean value of the NSS data, and peak points greater than the threshold are considered valid peak points.

However, the height information of the target cannot be obtained from the NSS, and a magnetic tensor must be used to obtain the height of the target. Nara et al. [30] derived the relationship among the dipole position, magnetic field vector, and magnetic gradient tensor.

(6) $$\left[\begin{array}{ccc} G_{xx} & G_{xy} & G_{xz}\\ G_{yx} & G_{yy} & G_{yz}\\ G_{zx} & G_{zy} & G_{zz} \end{array}\right]\left[\begin{array}{c} r_{x}-r_{x0}\\ r_{y}-r_{y0}\\ r_{z}-r_{z0} \end{array}\right]=-3\left[\begin{array}{c} B_{x}-B_{x0}\\ B_{y}-B_{y0}\\ B_{z}-B_{z0} \end{array}\right]$$

where $\left(r_{x},\;r_{y},r_{z}\right)$ is the measurement point position; $\left(r_{x0},\;r_{y0},r_{z0}\right)$ is the dipole position; $\left(B_{x},\;B_{y},B_{z}\right)$ is the magnetic field vector; and $\left(B_{x0},\;B_{y0},B_{z0}\right)$ is the background field. In Equation (6), the background field, magnetic field vector, magnetic gradient tensor, and sensor position are considered known quantities, and only the magnetic dipole coordinates are unknown. The background field can also be obtained by premeasurement. The expression for the dipole position is as follows:

(7) $$\left[\begin{array}{c} r_{x0}\\ r_{y0}\\ r_{z0} \end{array}\right]=\left[\begin{array}{c} r_{x}\\ r_{y}\\ r_{z} \end{array}\right]+3{\left[\begin{array}{ccc} G_{xx} & G_{xy} & G_{xz}\\ G_{yx} & G_{yy} & G_{yz}\\ G_{zx} & G_{zy} & G_{zz} \end{array}\right]}^{-1}\left[\begin{array}{c} B_{x}-B_{x0}\\ B_{y}-B_{y0}\\ B_{z}-B_{z0} \end{array}\right]$$

With the horizontal position of the target deduced, 5 × 5 measurement grid data points were selected near each target position for rough position inversion. A 3D coordinate position point cloud was obtained from the magnetic tensor inversion of these grid points, and the k-means clustering algorithm was applied to calculate the cluster centres of the point cloud clusters to obtain a rough estimate of the height of each target.

### 2.4. Precise Positioning Based on TRR Optimization Algorithms

After roughly estimating the positions of multiple targets, an optimization algorithm was used to further invert the exact parameters of the multiple targets. In conventional magnetic target localization methods, owing to the large search range of target parameters, heuristic optimization algorithms are generally combined with optimization methods such as LM to achieve a balance between optimization capability and solution speed. In contrast, the multistep inversion method adopted in this study can provide a rough estimation of the multi-objective parameters, which replaces the function of the conventional heuristic optimization algorithm. Therefore, the subsequent precise localization part can use a faster local optimization method to invert the parameters.

The problem of parameter estimation for multiple dipoles can be formulated as follows:

(8) $$\begin{array}{l} X^{*}=\underset{X}{argmin}E\left(X\right)\\ X=[x_{1},\;y_{1},\;z_{1},\;M_{1},\;\theta_{1},\;\varphi_{1},\;\ldots \;,x_{N},\;y_{N},\;z_{N},\;M_{N},\;\theta_{N},\;\varphi_{N}] \end{array}$$

*X* is the parameter of *N* targets, the objective function *E* is the sum of the three-component errors between the measured and theoretical values of the magnetometer, *P* is the total number of sensors. $B_{k,x}^{\prime }$, $B_{k,y}^{\prime },\;B_{k,z}^{\prime }$ are the measured values of the triaxial magnetic field of the *k* sensor, and $B_{k,x}$, $B_{k,y},\;B_{k,z}$ are theoretically estimated values.

(9) $$E=\sum_{k=1}^{P}\left[{\left(B_{k,x}^{\prime }-B_{k,x}\right)}^{2}+{\left(B_{k,y}^{\prime }-B_{k,y}\right)}^{2}+{\left(B_{k,z}^{\prime }-B_{k,z}\right)}^{2}\right]$$

The trust region reflective (TRR) algorithm was used for the nonlinear least-squares problems. Compared with the traditional linear search algorithm, the TRR algorithm has the advantages of robustness and fast convergence.

The TRR algorithm divides the entire solution domain into several trust region subproblems by introducing a trust region. In each iteration process, according to the minimum value condition, a trial step s is obtained. The solution or the radius of the trust domain is updated through the iterative process until the error function satisfies the tolerance convergence condition E(X)<tol.

The trust domain algorithm deprecises an n-dimensional trust region $N_{i}$ around the current iteration position $x_{i}$. A neighbourhood of $N_{i}$ is deprecised by the current step size $s_{i}$, solution $x_{i}$ and the radius of the trust domain $r_{i}$ described in Equation (10).

(10) $$N_{i}=\left\{s_{i}\in P_{i}\;|\;∥s_{i}-x_{i}∥\leq r_{i}\right\}$$

Within this trust region, a simpler model function is used to ψ(x) to approximate the objective function f(x). This model function generally uses a second-order Taylor expansion form of the objective function. Other iterative algorithms are then used to solve the minima (subproblem) of the model function in this trust domain $\psi \left(x\right)$ as shown in Equation (11).

(11) $$\min_{s_{i}\in N_{i}}\{\cdot \psi_{i}\left(s_{i}\right)=g_{i}^{T}s_{i}+\frac{1}{2}s_{i}^{T}H_{i}s_{i}\}$$

where $s_{i}$ is the vector form of the step size, and $g_{i}$ is the gradient of f(x), and $H_{i}$ is the Hessian matrix of f(x).

**Figure 6:**
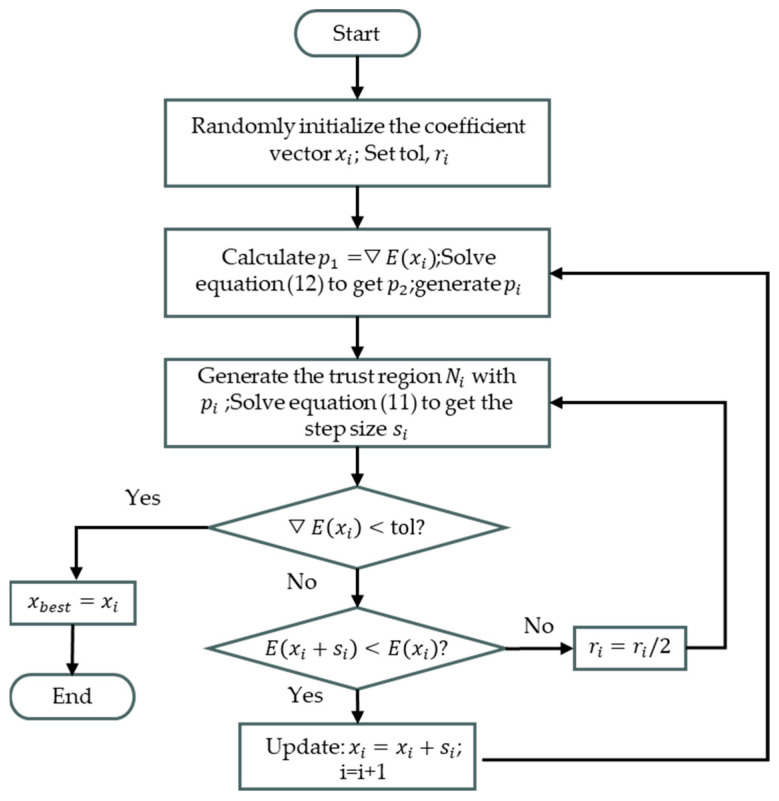
Flowchart for solving *X*_best_ by TRR algorithm.

The computational flowchart for determining the parameter *X* is shown in Figure 6.

To speed up the convergence of the algorithm, the $E(x_{i})$ the direction of the gradient of $▽E(x_{i})$, denoted as vector $p_{1}$, and the Gauss–Newton search direction, denoted as $p_{2}$ to generate the solution plane $P_{i}$. $p_{2}$ can be obtained by solving Equation (12).

(12) $$H_{i}p_{2}=-g_{i}$$

The ratio $r_{i}$ of the amount of change in the target function $f\left(x\right)$ to the amount of change in the model function $\psi \left(x\right)$ is used to evaluate the appropriateness of the current step size of the model function in the current domain of trust [31]. The ratio $r_{i}$ is deprecised as in Equation (13) shown.

(13) $$\begin{array}{l} r_{i}=\frac{\Delta f_{i}}{\Delta \psi_{i}}\\ \Delta \psi_{i}=\psi_{i}\left(0\right)-\psi_{i}\left(d\right)\\ \Delta f_{i}=f\left(x_{i}\right)-f\left(x_{i}+d\right) \end{array}$$

If $r_{i}$ is close to 1, indicating that the model function is very close to the objective function, then the current solution is $x_{i}$ is updated to $x_{i}+s_{i}.$ Otherwise, the current solution should be kept unchanged and the trust domain radius should be reduced. If the error function is less than the tolerance limit after iteration, stop iteration and output the parameter estimation results.

## 3. Results and Discussion

In this study, we employed a maximum of five NdFeB cylindrical magnets (N35) as magnetic sources, each possessing a total magnetic moment intensity spanning from 0.01 to 0.05 A·m^2^. Enhancing precision in determining the true positions of these magnetic sources, we devised a standardized experimental plate positioned atop the magnetometer array. This plate featured a marked scale for reference. Precise tuning of the distance between the magnetometer array and the experimental plate was achieved through a selection of hexagonal copper post units of varying lengths. A two-tiered platform was built above the sensor array to simulate different heights.

**Figure 7:**
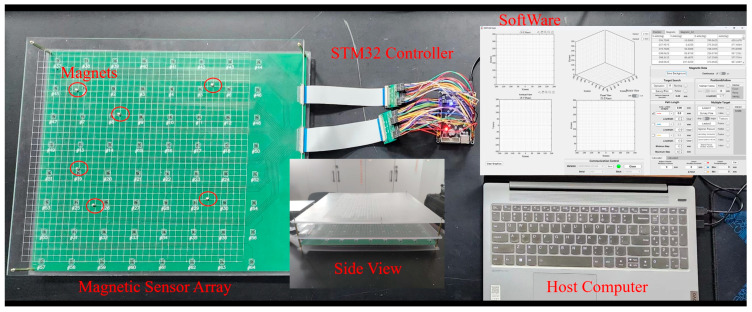
Magnetic sensor array and data acquisition system. The red circle shows the placed magnets.

As shown in Figure 7, 64 magnetometers (MMC8593A) are distributed on a 350 mm × 350 mm printed circuit board. The chip can measure magnetic fields of up to 8 G with a noise level of 0.4 mG RMS. The magnetic flux density measured by the magnetometers was captured by the STM32 board before being transferred to a computer, and the algorithm calculated the 6D parameters of each magnetic target. The parameter settings of the magnetic positioning system and the positioning results are displayed on the graphical user interface of the computer. The following experiments were conducted without shielding from the electromagnetic field.

### 3.1. Super-Resolution Network Training and Testing

The training data used for the super-resolution neural network was a dataset created through simulation. We used low-resolution data as the network input and high-resolution data as the network output. Low-resolution (LR) data were obtained from an 8 × 8 sensor array with a distance of 5 cm between the sensors. High-resolution (HR) data were obtained using a 64 × 64 sensor array with a spacing of 0.556 cm. Magnetic moments M in the data set ranged from 0.01 to 0.05 A·m^2^. One to six magnetic dipoles were placed in a vertical range of 3 to 10 cm above the sensor array. The dipoles varied in size and orientation. The far-field equations for the magnetic dipoles were used to calculate the vector magnetic field generated at the corresponding sensor location, and Gaussian white noise with a standard deviation of 40 nT was added to the low-resolution data. The final dataset comprised 6 × 10^4^ records. Of these, 5 × 10^4^ formed the training set, and the remaining 10,000 were the test set. Here, the training set containing 5 × 10^4^ pieces of data is divided into five disjoint data subsets, and then one of the data subsets (1 × 10^4^ pieces) is sequentially used as the validation set, and the rest of the data subsets are used as the training set to train the model, and finally, the model with the smallest test error is selected as the final model. The specific dataset parameters are shown in Table 1. For each record, the sensor array provided magnetic field data along the three axes. Thus, the network input consisted of three channels of 8 × 8 low-resolution data, and the output consisted of three channels of 64 × 64 high-resolution data.

As shown in Equation (14), both the input and labelled data are normalized using the inverse-tangent method.

(14) $$B_{normalized}=\mathrm{atan}\left(k\cdot B\right)\cdot 2/\pi$$

where the units of the magnetic field data are Tesla (T) and the coefficient *k* is 2× 10^−5^. The normalization process rearranges magnetic field anomalies spanning multiple orders of magnitude into a more homogeneous distribution in the interval (−1, 1).

The network was implemented in PyTorch-Lightning 1.6.0 and Python 3.9 for training and inference, respectively. All the experiments were performed on a Windows 10 PC with an AMD Core 3900X CPU, 96 G RAM, and Nvidia GeForce RTX 3090 GPU (Nvidia CUDA 11.7).

In the training phase of this network, the network was optimized for the training process using the Adam optimizer for a total of 2 × 10^5^ iterations, where the initial learning rate was set to lr = 0.001, *β*_1_ = 0.9, and *β*_2_ = 0.9, with only L1 loss. We also tuned the models using 5-fold cross-validation, fully trained the best-performing models in the validation set, and saved them for subsequent testing and comparison.

In this study, we compared the proposed method with five SR reconstruction algorithms: Bicubic, EDSR [24], SAN [25], RCAN [26], and SwinIR [28]. The parameter settings for these networks refer to those proposed in the original study. In the NAFSR, each chunk contains 16 NAF blocks, and the number of convolutional channels within each NAF block is 64. We employ three metrics to assess the credibility of the inversion outcomes: structural similarity (SSIM), peak signal-to-noise ratio (PSNR), and mean absolute error (MAE). SSIM assesses the similarity of magnetograms in terms of magnitude and structure. PSNR, a prevalent objective evaluation index, quantifies error in pixel correspondence. Meanwhile, MAE represents the average absolute prediction error, offering a nuanced portrayal of accuracy. Table 2 presents the results..

Table 2 shows that the PSNR of the NAFSR was at least 1.69 dB higher than that of the other methods, and the MAE of the three axial directions was at least 4.56 nT lower. This indicates that the method used in this study has a clear advantage for the ×8 dataset. Considering the difference between magnetic field data and natural images, the above metrics were calculated using magnetic field data output values rather than image pixel values. Therefore, the corresponding calculated results may differ from typical values in conventional image processing fields.

**Figure 8:**
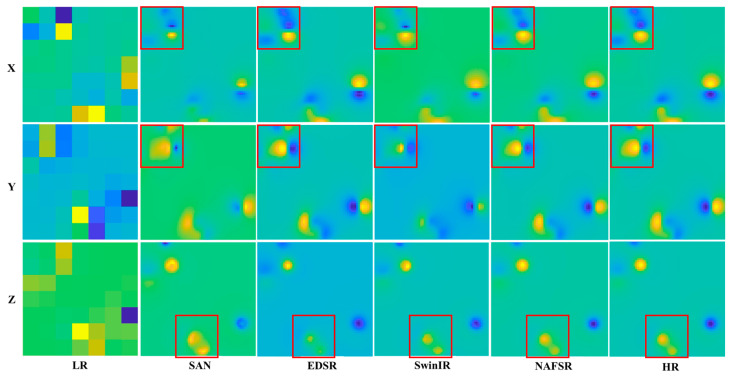
Subjective comparison of reconstruction results of different algorithms on ×8 dataset. The different colors represent the magnitude of the total magnetic induction. The red box shows the shape of the magnetic anomaly reconstructed by different algorithms.

In addition, a subjective analysis of the dataset (Figure 8) also revealed the superior performance of the proposed method. Comparing the red magnetic anomaly areas of the results of different algorithms, other SR algorithms usually produce blurred magnetograms due to underutilisation of features, whereas the method in this study produces magnetograms with more accurate and detailed textures. This qualitative improvement over other SR methods is also supported by the superior objective metrics.

The magnetograms obtained after super-resolution enhancement still have some residual errors compared to the real high-resolution magnetograms. This error may have been caused by two factors. The first is the fitting bias of the neural network itself. Second, the Gaussian white noise added to the input training data introduces errors. Because the magnitude of magnetic anomalies in this task is generally in the order of thousands of nT, the residual bias in the super-resolution results has little effect on subsequent localization. Overall, the network performed the super-resolution task well for the sparse magnetic data.

### 3.2. Multi-Targets Inversion in Field Tests

Simultaneous inversion tests for the five targets were performed using a magnetic sensor array, as shown in Figure 7. The prearranged positions and magnitudes of the magnetic moments of magnetic sources one to five are listed at the top of Table 3. The true magnetic moments of the targets were estimated by placing the targets to be measured individually on top of the array, and the data were acquired using the PSO-LM algorithm to calculate the magnetic moments of the individual targets.

**Figure 9:**
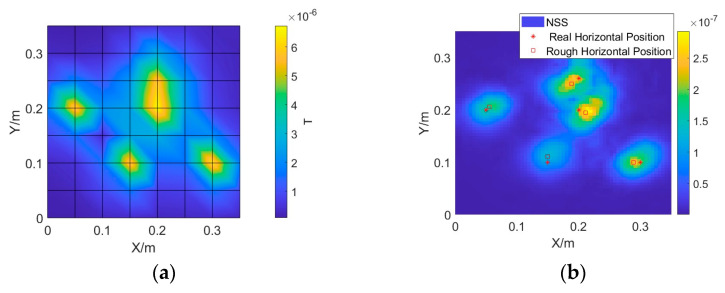
(**a**) Measured magnitude of magnetic anomaly caused by magnetic sources. (**b**) Calculated NSS using the SR magnetic vector data and estimated horizontal location.

The total field of the measured magnetic anomalies is shown in Figure 9a. Only four magnetic anomalies were observed. The magnetic anomalies produced by the two sources in the upper-right corner are superimposed. The NSS after super-resolution enhancement is shown in Figure 9b. Five magnetic anomalies are clearly observed in Figure 9b. By searching for the local maxima of the NSS, the horizontal coordinates of the sources can be obtained and labelled, as shown in Figure 9b. The approximately estimated horizontal coordinates are a little far from the real coordinates, but they can significantly constrain the search range for the next optimization algorithm.

**Figure 10:**
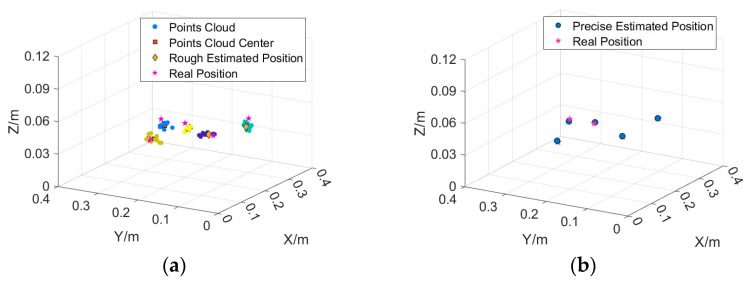
(**a**) rough estimated position and real position. (**b**) precise estimated position using TRR and real position. The spherical dots in the figure represent rough localisation point clouds, which are colored differently near different targets.

Several measurement points were selected at the predicted horizontal position of each target to calculate the tensor estimates. The positional point cloud of each target was then obtained based on the Nara method inversion, as shown in Figure 10a. The location of the centre of the point cloud obtained by Kmeans clustering is marked using boxes. The height of each target corresponding to the centre of the point cloud was the estimated height of the target. Thus, an approximate 3D estimated position of each target was obtained. From Figure 10a, it can be observed that there is a deviation in the rough estimation of the height direction for some targets.

The exact magnetic source position and magnetic moment parameters were estimated using the TRR algorithm based on the roughly estimated 3D data. The initial position values of the TRR algorithm were used to roughly estimate the 3D position. The magnetic moment parameters *M*, *θ*, and *φ* were randomly initialized in the range of [0.01, 0.05] A·m^2^, [−90°, 90°], and [−180°, 180°], respectively. The position search range is ±0.02 m around the roughly estimated position, and the magnetic moment parameter search range is the whole feasible domain. The results of the final exact inversion are presented in the lower part of Table 3. Figure 10 illustrates the distribution of the final estimated target and true positions. In this example, both the estimated magnetic source position and magnetic moment were closer to the true values. The positions of five magnetic sources were recovered using the proposed method with an estimation error of less than 3 mm in each axial direction. The method was also successful in estimating the magnitude of the magnetic moments, with a deviation of approximately 5% in the estimation of the moments for the three targets with larger moments and approximately 13% in the estimation of the moments for the two targets with smaller moments.

The horizontal positioning error in this positioning test is related to the super-resolution reconstruction error of the magnetic vector. The peak position indicated by super-resolution magnetic data sometimes does not exactly correspond to the true horizontal position. The positioning error in the vertical direction is also related to the magnetic field reconstruction error. This error also affects the accuracy of the magnetic tensor calculation, because the magnetic tensor is calculated from the super-resolution magnetic vector. However, overall, the rough positioning results of the target were close to the true position, which significantly reduced the search range of the 3D position.

The PSO-LM and WO-LM algorithms are widely used in applications where multiple magnetic targets are located. Therefore, the proposed SRMGT-TRR algorithm is compared with the PSO-LM algorithm and an improved IMWO-LM algorithm.The population size and the maximum number of iterations for the PSO algorithm are set to 100 and 50, respectively.The acceleration constants (c1, c2) are set to 1.49. The number of search agents and the maximum number of iterations for the IMWOA are set to 100 and 50, respectively. In practical experiments, if the global search range is used, it is difficult for the above two heuristics to achieve localization of 4–5 targets. Therefore, when using the heuristic methods for inversion, the approximate range of targets was roughly delineated according to the size of magnetic anomalies at different measurement points, which was ±0.05 m near the suspected coordinates in the horizontal direction and 0.02–0.1 m in the vertical direction.

The magnetic field values generated by ten sets of targets with different positions and magnetic moment parameters were collected using an 8 × 8 magnetic sensor array. As shown in Table 4, three groups had three targets, three groups had four targets, and four groups had five targets. The test samples included different magnetic moments and heights.

**Figure 11:**
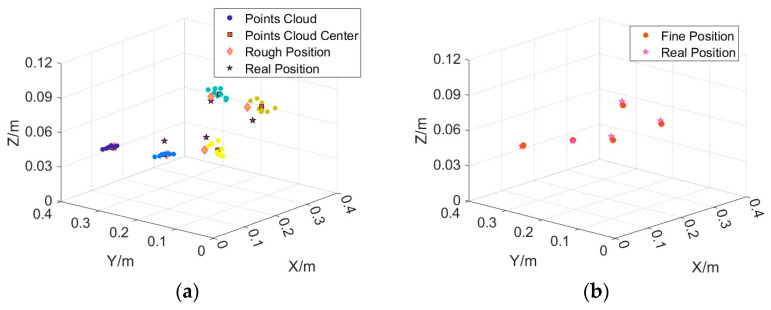
(**a**) rough estimated position and real position of the 10th group. (**b**) precise estimated position using TRR and real position of 10th group. The spherical dots in the figure represent rough localisation point clouds, which are colored differently near different targets.

The five targets in group 10 are distributed at two heights of 5 cm and 7 cm. The rough and precise localisation results for this group are shown in Figure 11a,b, with an average localisation error of 2.4 mm. the final precise localisation results show that the method is able to accurately target different planes.

**Figure 12:**
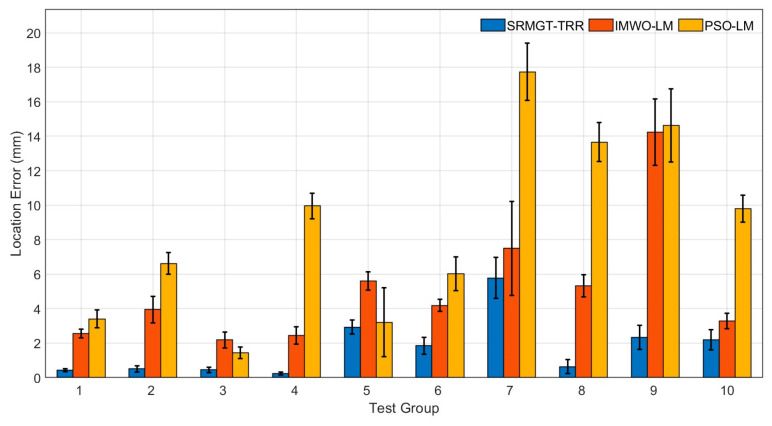
Positioning error of the proposed SRMGT-TRR algorithm, IMWO-LM algorithm, and PSO-LM algorithm.

**Figure 13:**
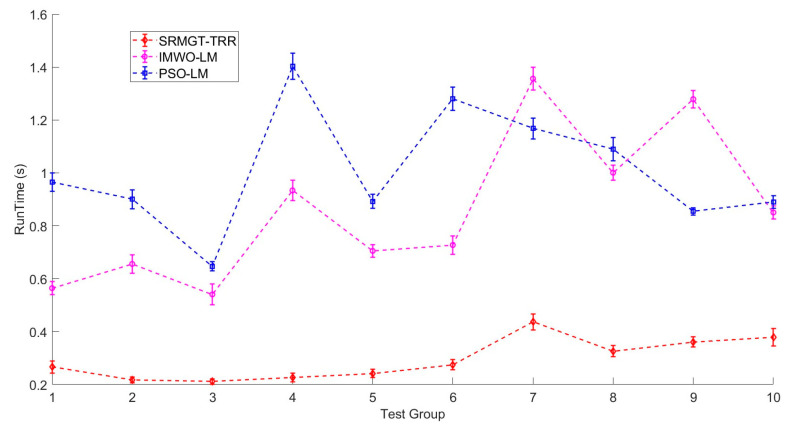
Running time of the proposed SRMGT-TRR algorithm, IMWO-LM algorithm, and PSO-LM algorithm.

Each algorithm was run 100 times for each test sample to determine the average localization error, magnetic moment inversion error, and running time. Calculations were performed using the computer on which the super-resolution neural network was trained. The positioning accuracy and runtime results of the experiments with ten sets of targets are shown in Figure 12 and Figure 13, respectively.

As shown in Figure 12, the 3D positioning accuracies of the proposed SRMGT-TRR, PSO-LM, and IMWO-LM algorithms were different in the 10 groups with 100 positioning times. The average positioning errors (Table 5) of the PSO-LM algorithm, IMWO-LM algorithm and proposed SRMGT-TRR algorithm in multiple positioning were 8.64 mm, 5.13 mm, and 1.83 mm, respectively, and the average magnetic moment inversion errors were 9.70%, 8.23%, and 6.29%, respectively. The size of the test board here is 350 mm × 350 mm. When the number of targets increased, the average positioning errors of the three algorithms increased by different degrees. Compared to the PSO-LM and IMWO-LM algorithms, the proposed SRMGT-TRR method exhibited better performance in multitarget localization and magnetic moment inversion accuracy.

Additionally, we compared the execution times of the proposed SRMGT-TRR, PSO-LM, and IMWO-LM algorithms. As shown in Figure 13, the average running times of the three algorithms are marked using diamonds, squares, and circles, and each data point represents the average time of 100 localizations. The average time required by the SRMGT-TRR algorithm proposed in this study was 0.294 s, whereas the average times required by the PSO-LM and IMWO-LM algorithms were 1.009 and 0.861 s, respectively. The PSO-LM and IMWO-LM algorithms are slower because of the presence of a random selection process with an uncertain time. In the proposed SRMGT-TRR algorithm, the average time of the inference process of the super-resolution neural network was 49 ms, the average time of rough localization was 30 ms, and the average time of precise localization was 215 ms. The computation time of the proposed SRMGT-TRR algorithm was significantly shorter than that of the other two methods. Therefore, the proposed SRMGT-TRR algorithm meets the requirements for real-time effective localization of multiple magnetic targets.

In this experiment, the running times of PSO-LM and IMWO-LM were longer than those in [14,17]. In addition to the effects of equipment configuration and hyperparameter settings, the more important reason is that the arrays in this study are of 8 × 8 size, whereas in [14,17], the size is 3 × 3. A large array must calculate the magnetic-field values of more measurement points when calculating the magnetic field, which leads to different computing times. However, the average computation time of the method proposed in this paper is still lower than 300 ms for large arrays, which proves that it still has the ability for real-time inversion when the array size is extended.

### 3.3. Rough Positioning Performance Evaluation

The rough localisation step determines the number of targets and the rough location of targets by searching for NSS peaks. The results of this step have a strong influence on the precise localisation. Therefore, the performance of this step in determining the number of targets and rough localisation is evaluated through several simulation experiments. The rough localisation algorithm processes the 6000 test sets generated based on the simulation parameters in Table 2 and outputs the predicted number of targets and target rough locations. The number of targets range from one to six. This is to evaluate the performance of the algorithm for larger number of targets. 

**Figure 14:**
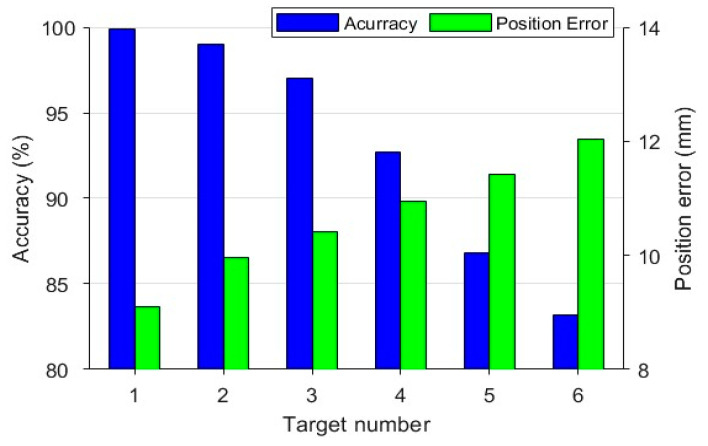
Accuracy of target number estimation and rough positioning error.

By comparing with the set target correct parameters, the quantity prediction accuracy and the rough estimated position deviation can be obtained, as shown in Figure 14. The positional error is only calculated for those with correct quantity estimates.

When the number of targets is less than or equal to four, the accuracy of target number prediction is higher than 90%, and the average rough positioning error is not more than 11mm, but when the number of targets is six, the accuracy of target number prediction is less than 85%, and the rough positioning error reaches 12 mm. the rough positioning result is poorer when the number of targets is more than five, which is a limitation in practical applications.

There are two reasons why rough localisation performance decreases when the number of targets increases. One is that the output of the super-resolution neural network may deteriorate when the number of targets increases. The second is that the magnetic fields generated when multiple targets are close to each other interfere with each other, resulting in some of the peaks being difficult to identify or incorrectly identified.

### 3.4. End-to-End Inversion Performance Evaluation

**Figure 15:**
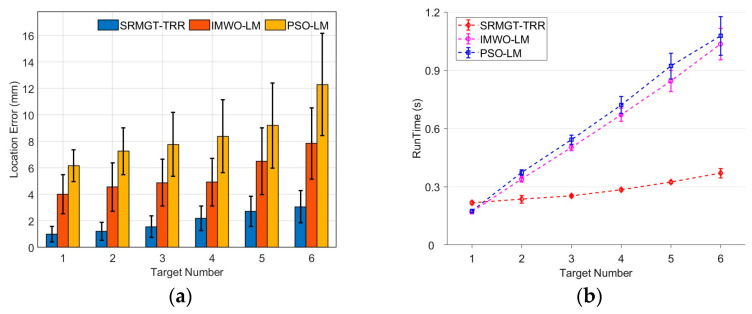
(**a**) End-to-end positioning error and (**b**) running time of the proposed SRMGT-TRR algorithm, IMWO-LM algorithm, and PSO-LM algorithm.

Multiple end-to-end repetitive tests are performed on the three localisation algorithms to evaluate the localisation accuracy, runtime and localisation success of the different methods. Since it is difficult to obtain large quantities of data from real experiments, the results are obtained through simulation experiments. Similar to the rough positioning performance evaluation, end-to-end inversion calculations are performed using 6000 test data for one to six targets. In this case, the number of entries for each target quantity is 1000. In order for PSO-LM and IMWO-LM to converge properly for conditions larger than three targets, the same rough initial values are provided to limit the search range. The positioning errors and running times of different algorithms at a different number of targets are statistically obtained as shown in Figure 15.

The average localisation error of SRMGT-TRR method is less than 3 mm for different number of targets, which is better than the other two algorithms. The computation times of the three methods are close to each other in the single target case. However, the average positioning time of SRMGT-TRR is much smaller than the other two methods when the number of targets is large. In the case of one to five targets, the average positioning time is less than 300 ms.

**Figure 16:**
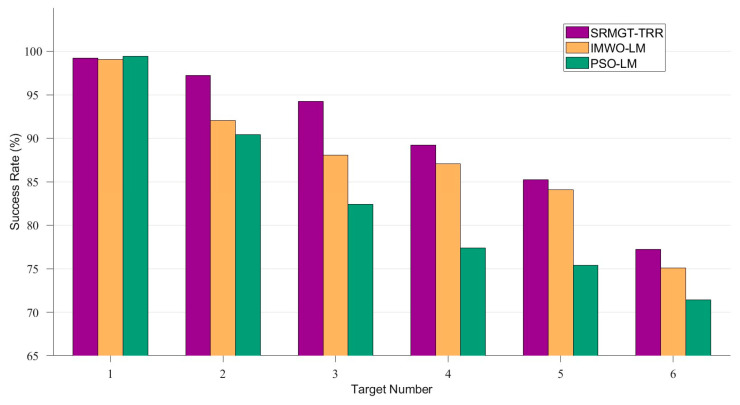
Success rate of the proposed SRMGT-TRR algorithm, IMWO-LM algorithm, and PSO-LM algorithm.

In practical applications, the localisation success rate is also an important indicator of system performance. In this paper, the localisation success rate is defined as the ratio of the number of samples whose localisation results are less than 2 cm away from the true position to the total number of samples. The positioning success rate of different algorithms is shown in Figure 16.

The results of multiple repeated experiments show that the end-to-end localisation success rate of different algorithms is inversely proportional to the number of targets. The SRMGT-TRR method proposed in this paper has a higher localisation success rate compared to the other methods.The localisation success rate of SRMGT-TRR is more than 85% for the number of targets from one to five. The success rates of all three methods are close to 100% for single-target localisation. When the number of targets is more than 5, the end-to-end localisation success rate of different algorithms is less than 80%. Therefore, the methods proposed in this paper have a high success rate when dealing with 1 to 5 targets, and the algorithm performance needs to be continued to be optimised for a larger number of targets.

In summary, super-resolution data enhancement and rough localization steps in the proposed method are more critical than precise localization. The quality of data enhancement determines the accuracy of rough localization. The rough localization results determine the quality of precise localization. The next step is to optimize the generalisation ability of the super-resolution network such that it can adapt to more complex magnetic environments. It is also necessary to investigate how to obtain more precise 3D positions by the inversion of super-resolution magnetic tensor data and directly invert reliable magnetic moments.

## 4. Conclusions

In this study, an innovative method for the rapid localization of an unknown number of multiple magnets based on magnetic sensor arrays was proposed. A super-resolution neural network was used to augment sparse magnetic array data into dense data. The number of targets and rough 3D positions were determined based on the NSS and magnetic tensor inversion of the dense data. Combining the rough position results, the TRR algorithm was applied to achieve a precise inversion of the multi-target parameters. Experiments based on 8 × 8 arrays demonstrate that the proposed method locates three to five targets with an average error of less than 3 mm and an average time of less than 300 ms. This indicates that the proposed method can quickly and accurately invert multiple targets and meets the requirements for real-time effective localization of multiple magnetic targets. It is expected that this method can achieve superior localisation results in situations where the number of targets is known and unknown, such as medical interventions and intrusion target detection.

## Figures and Tables

**Table 1:** Parameter ranges for generated data.

Parameter	Value Range	Parameter	Value Range
Target number	[1, 6]	*θ*/°	[−90, 90]
*x*/m	[0, 0.35]	*φ*/°	[−180, 180]
*y*/m	[0, 0.35]	*M*/A·m^2^	[0.01, 0.05]
*z*/m	[0.03, 0.1]	Training set	5 × 10^4^
Validating set	1 × 10^4^	Testing set	1 × 10^4^

**Table 2:** Comparison of reconstruction results from different algorithms under the ×8 dataset.

Algorithms	PSNR (dB)/SSIM/MAE (nT)		
	X	Y	Z
BiCubic	19.88/0.521/65.0	19.88/0.519/64.9	19.67/0.559/84.2
EDSR	38.99/0.987/12.1	39.14/0.988/15.5	39.81/0.990/21.2
SAN	36.74/0.989/39.2	36.69/0.989/40.6	37.41/0.991/58.9
RCAN	38.79/0.992/44.3	38.91/0.992/35.9	39.09/0.993/33.2
SwinIR	39.87/0.995/29.5	39.85/0.989/26.3	40.41/0.994/62.3
NAFSR	41.56/0.996/8.1	42.90/0.995/6.4	43.37/0.996/20.6

**Table 3:** Prearranged locations, estimated locations, and moments of the magnetic sources.

	No.	*x*/m	*y*/m	*z*/m	*θ*/°	*φ*/°	*M*/A·m^2^
Prearranged	1	0.05	0.30	0.05	-	-	0.015
	2	0.15	0.25	0.05	-	-	0.02
	3	0.30	0.25	0.05	-	-	0.02
	4	0.10	0.10	0.05	-	-	0.02
	5	0.25	0.10	0.05	-	-	0.015
Estimated	1	0.051	0.301	0.052	−87.41	−180.00	0.017
	2	0.151	0.251	0.049	87.30	42.91	0.021
	3	0.301	0.251	0.049	87.89	−21.17	0.021
	4	0.100	0.101	0.050	84.91	30.10	0.021
	5	0.249	0.101	0.049	88.92	−180.00	0.017

**Table 4:** List of 10-parameter groups.

No.	3D Position (cm) & Total Magnetic Moment (A·m^2^)				
	Target 1	Target 2	Target 3	Target 4	Target 5
1	(10, 25, 5), 0.02	(25, 25, 5), 0.02	(18, 12, 5), 0.02	/	/
2	(15, 22, 5), 0.02	(18, 12, 5), 0.02	(29, 22, 5), 0.02	/	/
3	(10, 20, 5), 0.02	(20, 10, 5), 0.02	(25, 20, 5), 0.01	/	/
4	(10, 30, 5), 0.02	(20, 25, 5), 0.02	(15, 15, 5), 0.02	(25, 10, 5), 0.02	/
5	(10, 25, 5), 0.02	(25, 25, 5), 0.02	(15, 10, 5), 0.02	(30 10, 5), 0.02	/
6	(10, 25, 5), 0.02	(25, 25, 5), 0.01	(15, 10, 5), 0.02	(30, 10, 5), 0.01	/
7	(10, 25, 5), 0.02	(20, 25, 5), 0.02	(30, 25, 5), 0.02	(20, 15, 5), 0.02	(20, 5, 5), 0.02
8	(5, 20, 5), 0.02	(20, 26, 5), 0.02	(20, 20, 5), 0.02	(15, 10, 5), 0.02	(30, 15, 5), 0.02
9	(5, 30, 5), 0.015	(15, 25, 5), 0.02	(30, 25, 5), 0.02	(10, 10, 5), 0.02	(25, 10, 5), 0.015
10	(5, 30, 5), 0.015	(15, 25, 5), 0.02	(30, 25, 7), 0.02	(10, 10, 7), 0.02	(25, 10, 7), 0.015

**Table 5:** Estimated error of position and magnetic moment and running time of three algorithms.

	Position Error (mm)	Magnetic Moment Error (%)	Running Time (s)
PSO-LM	8.64	9.70	1.009
IMWO-LM	5.13	8.23	0.861
SRMGT-TRR	1.83	6.29	0.294

## Data Availability

Data are contained within the article.

## References

[1] Su S., Zeng X., Song S., Lin M., Dai H., Yang W., Hu C. (2020). Positioning Accuracy Improvement of Automated Guided Vehicles Based on a Novel Magnetic Tracking Approach. IEEE Intell. Transp. Syst. Mag..

[2] Tian Z.X. Underwater Magnetic Surveillance System for Port Protection. Proceedings of the 2011 IEEE 2nd International Conference on Computing, Control and Industrial Engineering.

[3] Ma Y., Mao Z.-H., Jia W., Li C., Yang J., Sun M. (2011). Magnetic Hand Tracking for Human-Computer Interface. IEEE Trans. Magn..

[4] Sebkhi N., Bhavsar A., Anderson D.V., Wang J., Inan O.T. (2021). Inertial Measurements for Tongue Motion Tracking Based on Magnetic Localization With Orientation Compensation. IEEE Sens. J..

[5] Baldoni J.A., Yellen B.B. (2007). Magnetic Tracking System: Monitoring Heart Valve Prostheses. IEEE Trans. Magn..

[6] Wang M., Song S., Liu J., Meng M.Q.-H. (2021). Multipoint Simultaneous Tracking of Wireless Capsule Endoscope Using Magnetic Sensor Array. IEEE Trans. Instrum. Meas..

[7] Song S., Wang S., Yuan S., Wang J., Liu W., Meng M.Q.-H. (2021). Magnetic Tracking of Wireless Capsule Endoscope in Mobile Setup Based on Differential Signals. IEEE Trans. Instrum. Meas..

[8] Tiryaki M.E., Elmacıoğlu Y.G., Sitti M. (2023). Magnetic Guidewire Steering at Ultrahigh Magnetic Fields. Sci. Adv..

[9] Chen S., Zhang S., Luan X., Liu Z. (2020). Improved Differential Evolution Algorithm for Multi-Target Response Inversion Detected by a Portable Transient Electromagnetic Sensor. IEEE Access.

[10] McFee J., Das Y. (1981). Determination of the Parameters of a Dipole by Measurement of Its Magnetic Field. IEEE Trans. Antennas Propagat..

[11] Ge H., Song S., Wang J., Meng M.Q.-H. Multi-Magnet Tracking Method Using Extended Kalman Filter. Proceedings of the 2021 IEEE Sensors.

[12] Chen S., Zhu M., Zhang Q., Cai X., Bo X. Accurate Magnetic Object Localization Using Artificial Neural Network. Proceedings of the 2019 15th International Conference on Mobile Ad-Hoc and Sensor Networks (MSN).

[13] Liang K., Xie H., Wang G. Multiple Underwater Magnetic Targets Location Method Based on Neural Network. Proceedings of the 2021 4th International Conference on Advanced Electronic Materials, Computers and Software Engineering (AEMCSE).

[14] Song S., Hu C., Li M., Yang W., Meng M.Q.-H. Two-Magnet-Based 6D-Localization and Orientation for Wireless Capsule Endoscope. Proceedings of the 2009 IEEE International Conference on Robotics and Biomimetics (ROBIO).

[15] Yang W., Hu C., Li M., Meng M.Q.-H., Song S. (2010). A New Tracking System for Three Magnetic Objectives. IEEE Trans. Magn..

[16] Taylor C.R., Abramson H.G., Herr H.M. (2019). Low-Latency Tracking of Multiple Permanent Magnets. IEEE Sens. J..

[17] Lv B., Qin Y., Dai H., Su S. (2021). Improving Localization Success Rate of Three Magnetic Targets Using Individual Memory-Based WO-LM Algorithm. IEEE Sens. J..

[18] Chang S., Lin Y., Zheng Y.R., Fu X. (2020). Simultaneous Detection of Multiple Magnetic Dipole Sources. IEEE Trans. Magn..

[19] Li Q., Li Z., Shi Z., Fan H. (2022). Application of Helbig Integrals to Magnetic Gradient Tensor Multi-Target Detection. Measurement.

[20] Ding X., Li Y., Luo M., Chen J., Li Z., Liu H. (2022). Estimating Locations and Moments of Multiple Dipole-Like Magnetic Sources From Magnetic Gradient Tensor Data Using Differential Evolution. IEEE Trans. Geosci. Remote Sens..

[21] Li Q., Li Z., Shi Z., Fan H. (2022). Multi-Target Magnetic Positioning Using SAFCM Clustering and Invariants-Improved Tilt Angle. IEEE Trans. Geosci. Remote Sens..

[22] Zheng X., Tian Y., Wang B. (2023). A Magnetic Gradient Tensor Based Method for UXO Detection on Movable Platform. IEEE Trans. Geosci. Remote Sens..

[23] Dong C., Loy C.C., He K., Tang X., Fleet D., Pajdla T., Schiele B., Tuytelaars T. (2014). Learning a Deep Convolutional Network for Image Super-Resolution. Computer Vision–ECCV 2014.

[24] Lim B., Son S., Kim H., Nah S., Lee K.M. Enhanced Deep Residual Networks for Single Image Super-Resolution. Proceedings of the 2017 IEEE Conference on Computer Vision and Pattern Recognition Workshops (CVPRW).

[25] Dai T., Cai J., Zhang Y., Xia S.-T., Zhang L. Second-Order Attention Network for Single Image Super-Resolution. Proceedings of the 2019 IEEE/CVF Conference on Computer Vision and Pattern Recognition (CVPR).

[26] Zhang Y., Li K., Li K., Wang L., Zhong B., Fu Y., Ferrari V., Hebert M., Sminchisescu C., Weiss Y. (2018). Image Super-Resolution Using Very Deep Residual Channel Attention Networks. Computer Vision—ECCV 2018.

[27] Chen X., Chu L. (2022). Simple baselines for image restoration. European Conference on Computer Vision.

[28] Liang J., Cao J., Sun G., Zhang K., Van Gool L., Timofte R. SwinIR: Image Restoration Using Swin Transformer. Proceedings of the 2021 IEEE/CVF International Conference on Computer Vision Workshops (ICCVW).

[29] Hu S., Tang J., Ren Z., Chen C., Zhou C., Xiao X., Zhao T. (2019). Multiple Underwater Objects Localization With Magnetic Gradiometry. IEEE Geosci. Remote Sens. Lett..

[30] Nara T., Suzuki S., Ando S. (2006). A Closed-Form Formula for Magnetic Dipole Localization by Measurement of Its Magnetic Field and Spatial Gradients. IEEE Trans. Magn..

[31] Coleman T.F., Yuying L. (1996). An interior trust region approach for nonlinear minimization subject to bounds. SIAM J. Optim..

